# Effects of Chili Straw Biochar on Alfalfa (*Medicago sativa* L.) Seed Germination and Seedling Growth on Electrolytic Manganese Residue

**DOI:** 10.3390/plants14172635

**Published:** 2025-08-24

**Authors:** Yang Luo, Yangzhou Xiang, Jun Ren

**Affiliations:** School of Geography and Resources, Guizhou Education University, Guiyang 550018, China; luoyang@gznc.edu.cn (Y.L.); yzhxiang18@126.com (Y.X.)

**Keywords:** electrolytic manganese residue, alfalfa, physiological response, seed germination, nutrients, seedling growth

## Abstract

This study employed a pot experiment to compare the effects of varying application rates of chili straw biochar on seed germination and seedling growth of alfalfa (*Medicago sativa* L.) cultivated in electrolytic manganese residue (EMR) and to elucidate the underlying mechanisms. We aimed to provide a theoretical basis for vegetation restoration and manganese pollution control at EMR disposal sites. Our results indicated that while chili straw biochar did not affect the seed germination rate, it significantly enhanced the germination energy. In addition, treatment with 5% biochar significantly increased the germination index. Biochar application increased alfalfa seedling height (6.13 cm in the control group vs. 6.63–7.20 cm in the treated groups). Concurrently, the aboveground fresh biomass significantly increased by 49–77% compared to the control. Additionally, biochar application elevated chlorophyll content and reduced malondialdehyde content in alfalfa leaves. Correlation analysis revealed that the primary mechanisms underlying biochar-mediated improvement in seed germination and seedling growth involved enhancing the organic matter, available nitrogen, and available phosphorus content in the EMR, while decreasing the available manganese content. Overall, the application of 5% biochar in EMR optimally improved alfalfa plant growth and development.

## 1. Introduction

Manganese (Mn) is a critical strategic resource extensively utilized in industries such as steel manufacturing, agriculture, chemical production, high-end equipment manufacturing, and advanced materials [1]. The predominant method for Mn smelting is the electrolytic process, which involves leaching divalent manganese ions (Mn^2+^) by adding sulfuric acid and liquid ammonia to manganese carbonate ore, followed by the electrochemical reduction of Mn^2+^ to elemental Mn. During this process, approximately 10–12 metric tons of electrolytic manganese residue (EMR) are generated per ton of metallic Mn produced [2]. Currently, the primary disposal methods for EMR include landfill deposition, open-air impoundment, and storage in anti-seepage residue ponds. As of 2023, the global stockpile of EMR has exceeded 160 million tons, with an annual increase of more than 10 million tons [3,4]. On the one hand, the stockpiling of EMR occupies a large amount of land, reduces biodiversity, and damages the ecological landscape of the affected regions. On the other hand, the high Mn content in EMR can migrate to the surrounding soil, surface water, and groundwater during weathering, causing environmental pollution [5]. Therefore, implementing vegetation restoration on EMR disposal sites and controlling Mn migration from the residue are urgently warranted for ecological conservation and sustainable development.

Extensive research has demonstrated that the application of soil amendments can effectively enhance the properties of plant growth substrates to promote seed germination and facilitate plant growth and development [6,7,8], thereby accelerating the vegetation restoration process in mining wastelands. Biochar, a solid material produced via the pyrolysis of biomass under oxygen-limited conditions, exhibits an alkaline nature, high specific surface area, abundant functional groups, and a porous structure. Enriched with essential nutrients, such as nitrogen, phosphorus, and potassium, it can ameliorate substrate physicochemical properties and immobilize heavy metals. Consequently, biochar has gained widespread attention as a soil amendment agent in recent research on vegetation restoration in mining wastelands and heavy metal remediation [9,10,11]. The effects of biochar in this context vary significantly in practical applications due to variations in raw material types and application rates. Chili (*Capsicum annuum* L.) is one of the most extensively cultivated vegetable crops in China, playing a pivotal role in driving crop diversification and enhancing farmers’ income [12]. Chili generates substantial straw during harvesting [13,14]. Chili straw residues are lignin-rich [15] and contain essential nutrients for plant growth. Converting these residues into biochar for use in amending plant growth substrates and remediating environmental contamination potentially represents an efficient and environmentally benign approach. However, studies on this topic remain limited, and the optimal application rates of chili straw biochar under varying experimental conditions are yet to be elucidated.

Alfalfa (*Medicago sativa* L.), one of the most extensively cultivated perennial forage legumes worldwide, delivers notable socioeconomic benefits [16]. Furthermore, alfalfa exhibits vigorous foliar growth and strong tolerance to heavy metals, coupled with resilience to cold, drought, salinity, and high temperatures [17]. Its high survival rate in diverse adverse environments and capacity for nitrogen fixation, which enhances substrate nutrition, establish this species as a pioneer plant for vegetation restoration in mining-degraded areas [18]. However, studies have documented that elevated Mn concentrations can still significantly suppress alfalfa growth [19,20]. Hence, reducing Mn bioavailability becomes imperative for cultivating alfalfa on EMR. Therefore, the current study employed EMR as a plant growth substrate and chili straw biochar as an amendment agent in a pot experiment. The objectives of our study included (1) screening the optimal application rate of chili straw biochar for promoting alfalfa growth on EMR; (2) investigating the physiological responses of alfalfa seedlings to chili straw biochar application; and (3) elucidating the mechanistic role of chili straw biochar in terms of manganese availability and nutrient dynamics in EMR.

## 2. Results

### 2.1. Effects of Chili Straw Biochar on Alfalfa Germination on EMR

Direct sowing of alfalfa seeds on EMR yielded a germination rate (GR) of 60.94%. Following amendment with varying levels of chili straw biochar, alfalfa germination rates ranged from 57.81% to 61.88% (Table 1), with no significant differences observed among the treatment groups (*p* > 0.05). The B2 group exhibited the highest germination energy (GE), followed by B1, B3, and control (CK, Table 1), with significant differences (*p* < 0.05) between the treated groups (B1–B3) and the CK group. Moreover, the B2 group exhibited significantly higher GE than the B1 group; however, the B2 and B3 groups exhibited comparable GE. The B2 group exhibited a germination index (GI) of 19.86 (Table 1), which was significantly higher than the GI of all other groups (*p* < 0.05). In addition, the CK, B1, and B3 groups exhibited comparable GI values (15.74, 16.76, and 17.13, respectively).

### 2.2. Effects of Chili Straw Biochar on Alfalfa Seedling Growth on EMR

**Figure 1:**
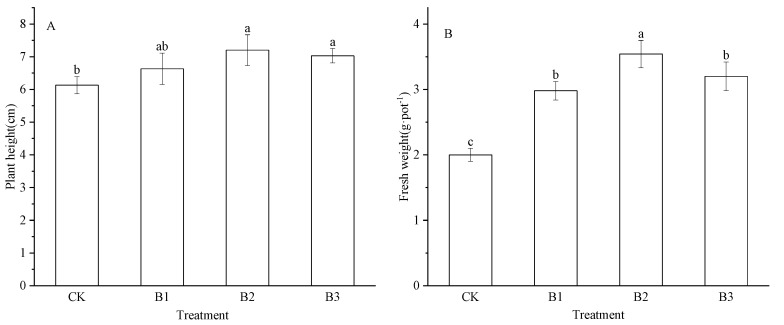
Plant height (**A**) (mean ± SD) and fresh weight (**B**) (mean ± SD) of alfalfa seedlings under different treatments. The bars with different lowercase letters indicate significant differences at *p* < 0.05. CK: Control treatment, B1: Treatment with 2.5% biochar, B2: Treatment with 5% biochar, B3: Treatment with 7.5% biochar.

As shown in Figure 1A, the average shoot heights of the CK, B1, B2, and B3 seedlings were 6.13, 6.63, 7.20, and 7.03 cm, respectively. The shoot height of the B2 and B3 seedlings differed significantly (*p* < 0.05) from the shoot height of CK seedlings. However, no significant differences were detected among the shoot heights of the treated groups. As shown in Figure 1B, the B1–B3 groups exhibited significantly higher (*p* < 0.05) shoot fresh weight than the CK group (49%, 77%, and 60% higher, respectively). Furthermore, only mild differences were observed between the shoot fresh weight of the B1 and B3 groups; however, the shoot fresh weight of these groups was significantly lower than that of the B2 group.

### 2.3. Effects of Chili Straw Biochar on Physiological Characteristics of Alfalfa Seedlings Cultivated on EMR

Table 2 lists the chlorophyll measurements from alfalfa seedling leaves. The B2 group exhibited the highest chlorophyll a concentration (2.02 mg·g^−1^), followed by the B3 (1.94 mg·g^−1^) and B1 (1.82 mg·g^−1^) groups. The levels in the B1 and B3 groups were comparable but significantly lower than those in the B2 group (*p* < 0.05). Furthermore, the treated groups contained 0.82–0.87 mg·g^−1^ chlorophyll b, with no significant intergroup differences among them. The CK group exhibited significantly lower chlorophyll a and b contents compared to the treated groups (1.46 and 0.57 mg·g^−1^). Variations in total chlorophyll content among the groups were similar to the trends in chlorophyll a. The B1, B2, and B3 groups exhibited significantly higher total chlorophyll contents compared to the CK group (30.05%, 42.36%, and 36.45% higher, respectively; *p* < 0.05). Furthermore, the B2 group exhibited significantly higher total chlorophyll levels compared to the B1 and B3 groups.

**Figure 2:**
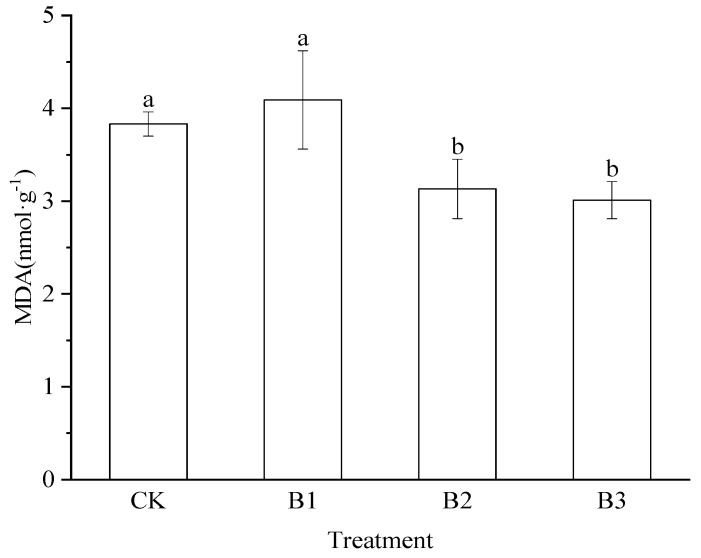
Malondialdehyde (MDA) content (mean ± SD) in alfalfa seedlings under different treatments. The bars with different lowercase letters indicate significant differences at *p* < 0.05. CK: Control treatment, B1: Treatment with 2.5% biochar, B2: Treatment with 5% biochar, B3: Treatment with 7.5% biochar.

MDA content of the groups ranged from 3.01 to 4.09 nmol/g (Figure 2). The MDA levels in the B1 group were comparable to those in the CK group but significantly higher than the levels in the B2 and B3 groups. The MDA levels in the B2 and B3 groups were comparable to each other but significantly lower than those in the CK group (*p* < 0.05).

### 2.4. Effects of Chili Straw Biochar on the Fundamental Properties of EMR

Table 3 lists the basic chemical properties of EMR under different treatments. The pH of EMR in the B1, B2, and B3 groups increased by 0.26, 0.48, and 0.7, respectively, compared to the control. Furthermore, the organic matter (OM) content of the EMR in the B1, B2, and B3 groups increased by 15.24%, 33.11%, and 46.27%, respectively, compared to the control, with significant differences among the treated groups (*p* < 0.05). All the treated groups exhibited significantly different alkali-hydrolyzable nitrogen (AN) levels (*p* < 0.05), demonstrating an initial increase followed by a decrease with rising biochar levels. The B2 group exhibited the highest AN levels (1.37-fold higher compared to the control). The available phosphorus (AP) levels in the B1 and B2 groups were comparable to each other but significantly higher than those in the CK and B3 groups. In addition, the available potassium (AK) levels of the treated groups ranged from 339.88 to 364.79 mg·kg^−1^, with no significant intergroup differences (*p* > 0.05).

**Figure 3:**
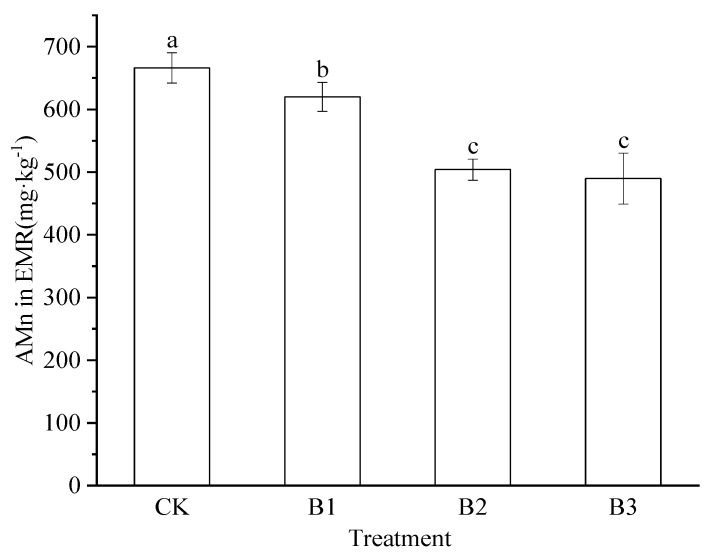
Available manganese (AMn) content (mean ± SD) in electrolytic manganese residue (EMR) under different treatments. The bars with different lowercase letters indicate significant differences at *p* < 0.05. CK: Control treatment, B1: Treatment with 2.5% biochar, B2: Treatment with 5% biochar, B3: Treatment with 7.5% biochar.

The available manganese (AMn) content in EMR decreased with increasing chili straw biochar levels, with the treated groups exhibiting significantly lower levels (reductions of 6.93% to 26.49%) compared to the control (*p* < 0.05, Figure 3). The highest and lowest AMn levels were observed in the CK (666.13 mg·kg^−1^) and B3 groups (489.68 mg·kg^−1^), respectively. Moreover, the AMn levels in the B2 and B3 groups differed only mildly from each other, but were significantly lower (reductions of 18.71% and 21.01%, respectively) compared to the levels in the B1 group.

### 2.5. Correlation Analysis Between EMR and Alfalfa Parameters

**Figure 4:**
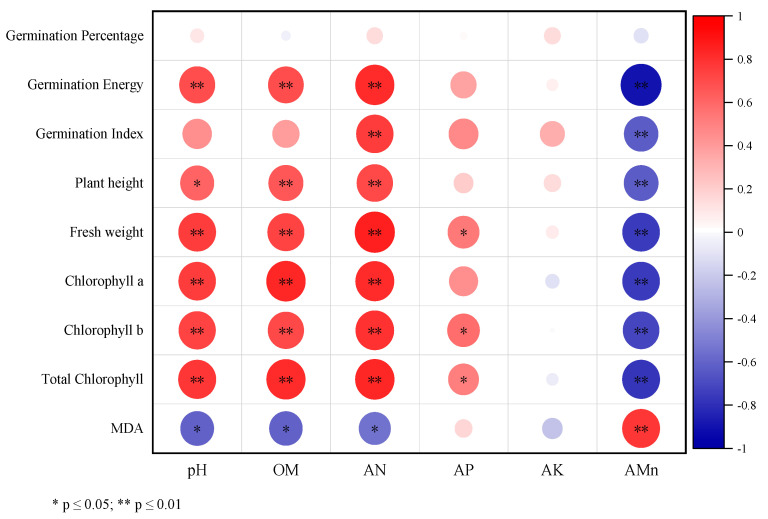
Pearson correlation heatmap between EMR indicators and alfalfa indicators. CK: Control treatment, B1: Treatment with 2.5% biochar, B2: Treatment with 5% biochar, B3: Treatment with 7.5% biochar. OM: Organic matter, AN: Alkali-hydrolyzable nitrogen, AP: Available phosphorus, AK: Available potassium. AMn: Available manganese. MDA: Malondialdehyde.

Pearson correlation analysis was performed between key chemical properties of EMR and growth or physiological parameters of alfalfa (Figure 4). Soil pH, OM content, and AN levels positively correlated (*p* < 0.01) with alfalfa GE, fresh weight, and chlorophyll content but negatively correlated (*p* < 0.05) with MDA content. Shoot height positively correlated with OM and AN levels (*p* < 0.01) and pH of EMR (*p* < 0.05). GI positively correlated with AN levels in EMR (*p* < 0.01). Furthermore, AP content in EMR positively correlated with alfalfa fresh weight, chlorophyll b, and total chlorophyll content. AMn content exhibited a significantly negative correlation with all alfalfa parameters (*p* < 0.01) except GR (no significant correlation), but was positively correlated with MDA content.

## 3. Discussion

### 3.1. Mechanisms Underlying the Impact of Chili Straw Biochar on the Chemical Properties and Manganese Availability of EMR

The acidity or alkalinity of plant growth substrates is primarily characterized by pH, a crucial chemical parameter of soil. In this study, the pH of EMR increased significantly with rising chili straw biochar levels, which was consistent with the results of previous studies [21]. This finding might be attributed to the alkaline functional groups on the biochar surface and the abundance of mineral-derived carbonates within its matrix. Upon incorporation into EMR, these alkaline components gradually release, neutralizing H^+^ ions in the residue and consequently elevating soil pH. Furthermore, base cations (for example, Ca^2+^, Mg^2+^, etc.) in biochar exchange with the acid-forming ions (for example, Al^3+^, H^+^, etc.) in EMR, thereby enhancing base saturation and increasing soil pH [22,23]. OM in cultivation substrates is an essential nutrient source for plant growth. Compared to the control, the EMRs treated with varying chili straw biochar levels exhibited 15.24–46.27% higher OM content. This result might be attributed to the substantial inherent organic carbon within biochar, which directly supplements the residue, thereby elevating OM levels [24]. The abundance and accessibility of available nutrients in cultivation substrates critically influence crop growth by impacting the availability of plant-usable nutrient forms. We observed that 2.5% and 5% chili straw biochar amendments significantly increased AN and AP content in EMR. This finding might be attributed to three factors: (1) decomposition of organic matter and minerals within biochar releases nitrogen, phosphorus, and other nutrients into EMR, thereby elevating its nutrient levels; (2) the intrinsic porous structure of biochar enhances nutrient adsorption and retention capacities of the soil, reducing leaching-related losses; and (3) biochar provides expanded microbial habitats and nutrient sources within EMR, accelerating microbe-mediated nutrient activation [25,26]. However, when chili straw biochar levels increased to 7.5%, AN and AP contents in EMR decreased. The nitrogen depletion might be attributed to the high C/N ratio (36.52:1) of the biochar, with excessive biochar application potentially inducing microbial immobilization of available nitrogen in the EMR to facilitate microbial proliferation [27]. The phosphorus depletion might be attributed to the increase in EMR alkalinity with increasing application rates of chili straw biochar. In this alkaline environment, soluble phosphate anions react with Ca^2+^ and Mg^2+^ to form sparingly soluble phosphate precipitates [28].

The available fraction of heavy metals represents the portion readily assimilated by plants, which more directly and accurately reflects their environmental and health risks. In the current study, the AMn content exhibited significantly negative correlations with alfalfa germination parameters and seedling growth, indicating its role as a limiting factor for plant growth on EMR. We observed significantly lower AMn content in biochar-amended EMR compared to the control group. This reduction is attributed to the extensive pore structure of EMR and abundant oxygen-containing functional groups in it, conferring it with a strong Mn adsorption capacity [29], thereby reducing its bioavailability. Concurrently, the alkaline constituents of chili straw biochar elevate the pH and carbonate content of EMR, facilitating precipitation of sparingly soluble manganese hydroxide (Mn(OH)_2_) and manganese carbonate (MnCO_3_) that inhibit Mn diffusion and mobility [30,31]. Notably, no significant difference was observed in the AMn contents of EMRs treated with 5% and 7.5% biochar, which might be attributed to a finite Mn adsorption capacity, with the saturation of surface sites at higher biochar application rates further diminishing Mn immobilization efficacy [32].

### 3.2. Mechanisms Underlying the Impact of Chili Straw Biochar on Alfalfa Seed Germination in EMR

Seed germination, the start of a plant’s life cycle, is highly sensitive to environmental conditions and crucial for subsequent plant growth. GR, the proportion of germinated seeds relative to the total number of seeds, is a key indicator of seed germination capacity. Gascó et al. [33] found that seed germination was not affected except for the inhibition caused by wood biochar in several species and the inhibition induced by paper sludge biochar on lettuce. After adding chili straw biochar, the GI of alfalfa seeds showed no significant change, which might be attributed to adequate support of the seed’s inherent nutrients to embryo development under optimal temperature and moisture conditions [34]. GE, the proportion of normally germinated seeds in the early phase, reflects germination speed and uniformity. Under Mn stress from EMR, the cell membrane structure and function of the seeds might be damaged due to the accumulation of reactive oxygen species (ROS) [35], impairing seed respiration and water absorption and leading to poor shoot-through-soil capability. However, after adding chili straw biochar, Mn bioactivity in the residue was suppressed, alleviating adverse conditions, accelerating germination, and thus enhancing alfalfa seed GE compared to the control. Wang et al. [36] found that the GI, which measures seed germination vigor, of the wheat seeds treated with 5% biochar was 15.43-fold higher compared to the control group, indicating effective alleviation of heavy metal stress. In line with these findings, we observed that alfalfa seeds in the B2 group (treatment with 5% chili straw biochar) showed the highest GI, suggesting that this biochar application rate was optimal for alfalfa seed germination and beneficial for subsequent seedling growth.

### 3.3. Mechanisms Underlying the Impact of Chili Straw Biochar on Alfalfa Seedling Growth in EMR

The seedling stage represents a critical and vulnerable phase in a plant’s life cycle, exhibiting high sensitivity to environmental factors. This stage directly influences a plant’s stress resistance, subsequent growth performance, and yield potential. Some studies suggest that adding an appropriate amount of biochar can alleviate the stress of heavy metals in soil, thereby promoting the growth of plant seedlings [37,38,39]. This study yielded similar results. The application of chili straw biochar resulted in a significant increase (49–77%) in the shoot fresh weight of alfalfa seedlings compared to the control group. This enhancement can be attributed to two primary mechanisms. On the one hand, chlorophyll is a vital pigment responsible for light harvesting and the absorption, transfer, and conversion of photosynthetic energy. However, Mn present in EMR can reduce chlorophyll synthesis by disrupting chloroplast ultrastructure. This phenomenon occurs when Mn^2+^ replaces Fe^2+^ and Mg^2+^ or binds to sulfhydryl groups (–SH) within chloroplast proteins [40]. The addition of chili straw biochar reduced Mn bioavailability in EMR, thereby impeding its uptake by alfalfa roots. Consequently, this mitigation of excess Mn levels alleviated its inhibitory effects on photosynthesis, promoting OM accumulation in the alfalfa plants. On the other hand, compared to typical agricultural soils, EMR possesses several detrimental characteristics, including low nutrient content, poor structure, and high AMn levels. Under such stressful conditions, plant antioxidant enzyme activity decreases and self-regulatory functions weaken. It leads to excessive ROS accumulation. This, in turn, causes severe cellular damage, leading to lipid peroxidation and the degradation of nucleic acids and other organic molecules, ultimately inhibiting plant growth and development [41,42]. Thus, the incorporation of chili straw biochar facilitated a comprehensive improvement of the internal environment of the EMR matrix, ameliorating the inherent adverse conditions. This was evidenced by a reduction in MDA content, a key marker of membrane lipid peroxidation, fostering the growth of alfalfa.

## 4. Materials and Methods

### 4.1. Test Materials

EMR was collected from the Changgou Section residue disposal site of Zunyi Manganese Mine in Guizhou Province, China, with an estimated stockpiling duration of 15–20 years. Extended exposure to biogeochemical weathering has induced incipient pedogenesis in the EMR, with observable but limited vascular plant establishment. Sampling sites were stratified based on topographic positioning. Composite samples (0–20 cm depth) from each site were obtained via “S”-path transects. Following field homogenization, residues were transported to the laboratory for use as potting substrates. After removing extraneous materials, the EMR was air-dried under laboratory conditions and sieved through a 5 mm nylon mesh for subsequent use. Its fundamental physicochemical properties and Mn content are presented in Table 4. Chili straw biochar was synthesized in a laboratory. Briefly, chili straw collected from the periphery of Wudang District, Guiyang City, Guizhou Province, China, was washed, oven-dried, and mechanically pulverized. The powdered material was transferred to ceramic crucibles, sealed, and pyrolyzed in a muffle furnace at 400 °C for 2 h. After cooling, the resulting biochar was sieved through a 100-mesh nylon screen and stored in sealed bags. The key characteristics of biochar are summarized in Table 4. The tested alfalfa variety was “Dite”, provided by Crowo (Beijing) Ecological Technology Co., Ltd. (Beijing, China). This cultivar demonstrated excellent agronomic performance in the Guizhou region. The seeds were surface sterilized by immersing them in 0.1% potassium permanganate solution for 15 min, followed by rinsing with sterile water and air-drying prior to experimentation.

### 4.2. Experimental Design

The experiment was conducted in a greenhouse at Guizhou Education University from June to September 2024. Based on methodologies established by Ai [43], Adams [44], and Arshad [45], chili straw biochar was thoroughly mixed with EMR at mass ratios of 2.5%, 5%, and 7.5%. The mixtures were transferred into plastic pots (16 cm diameter × 12 cm height), with 1 kg of mixture per pot, with biochar-free EMR serving as the control. Each treatment comprised four replicates, totaling 16 pots. Following a two-week equilibration period at room temperature, 80 alfalfa seeds were sown per pot.

During the plant growth phase, daily observations were recorded, and the pots were irrigated to maintain substrate moisture at approximately 60% of field capacity. Germination counts were monitored daily, with mold-compromised seeds promptly removed. Radicle emergence from the substrate constituted the germination criterion. The initiation of germination was defined as the date when the first seed germinated in each pot. Germination recording continued until one week after the stabilization of germination rates. Routine moisture management was followed until seedling harvest at 40 days post-sowing.

Then, the shoot biomass was rinsed with tap water, followed by deionized water, and blotted dry. Plant height and fresh weight were measured using a ruler and an analytical balance (with 0.0001 g precision), respectively. These samples were immediately subjected to physiological analyses. Simultaneously, EMR samples were homogenized and air-dried under laboratory conditions, and sieved after the removal of extraneous materials for subsequent determination of primary nutrients and available Mn content.

### 4.3. Measurement Items and Methods

#### 4.3.1. Seed GI

Seed germination was assessed using three parameters: GR, germination vigor, and GI. These parameters were calculated using the following formulas:

GR (%) = (m/N) × 100,

Germination vigor (%) = (n/N) × 100, and

GI = Σ(Gₜ/Dₜ)

Here, m: number of seeds germinated on day 7, N: total number of tested seeds, n: number of seeds germinated on day 3, Gₜ: number of seeds germinated at time t, and D: corresponding germination days at time t [46].

#### 4.3.2. Physiological Indicators of Seedlings

Chlorophyll was extracted using 95% ethanol, with absorbance measured at 649 nm (chlorophyll b) and 665 nm (chlorophyll a) for subsequent content calculation. MDA was quantified using the thiobarbituric acid colorimetric assay [43].

#### 4.3.3. Related Indicators of EMR

The pH of EMR was determined using a pH meter with a solid-to-water ratio of 1:2.5. OM content was quantified using the potassium dichromate volumetric method with external heating. AN content was measured via alkaline hydrolysis diffusion. AP was extracted with 0.5 mol L^−1^ NaHCO_3_ and determined via the molybdenum-antimony anti-spectrophotometric method. AK was extracted using NH_4_OAc and quantified by atomic absorption spectrophotometry [47]. AMn content in EMR was extracted with 0.1 mol L^−1^ HCl [48] and measured via atomic absorption spectrometry (novAA 350, Analytik Jena, Jena, Germany).

### 4.4. Data Processing

Data were preprocessed for means and standard deviations using Microsoft Excel 2019. One-way analysis of variance was conducted in IBM SPSS Statistics 22.0 to assess significant differences among treatments, followed by least significant difference LSD post-hoc testing for multiple comparisons. The relationships between alfalfa parameters and EMR properties were analyzed through Pearson correlation analysis. Figures were generated using OriginPro 2024.

## 5. Conclusions

Amendments with chili straw biochar enhanced OM content and improved nitrogen and phosphorus availability in EMR, thereby elevating its fertile status. Concurrently, biochar addition increased EMR pH while reducing AMn content, mitigating its toxic effects on alfalfa. Thus, chili straw biochar alleviated the adverse conditions imposed by EMR on plant development, accelerating alfalfa seed germination, promoting photosynthetic efficiency in leaves, and reducing membrane lipid peroxidation, consequently enhancing seedling growth. However, this study was primarily conducted under greenhouse conditions. The practical efficacy of chili straw biochar under field conditions requires validation. Concurrently, whether the nutritional quality (e.g., crude protein, crude fat, and fiber fractions) and heavy metal contents of alfalfa cultivated on EMR comply with relevant standards—and its suitability for livestock feeding—warrants further investigation. Additionally, future work could focus on chemically modifying chili straw biochar to enhance its Mn immobilization efficiency, thereby mitigating the environmental hazard of EMR.

## Figures and Tables

**Table 1:** Germination of alfalfa seeds under different treatments.

Treatment	GR (%)	GE (%)	GI
CK	60.94 ± 2.58 a	43.44 ± 2.77 c	15.74 ± 0.71 b
B1	57.81 ± 4.25 a	47.81 ± 2.77 b	16.76 ± 1.19 b
B2	61.88 ± 3.75 a	55.63 ± 2.98 a	19.86 ± 1.35 a
B3	60.00 ± 4.45 a	51.88 ± 2.17 ab	17.13 ± 0.93 b

Note: Each value represents the mean ± SD (standard deviation). Different lowercase letters within the same column indicate statistically significant differences among treatments (*p* < 0.05). GR: Germination rate, GE: Germination energy, GI: Germination index, CK: Control treatment, B1: Treatment with 2.5% biochar, B2: Treatment with 5% biochar, B3: Treatment with 7.5% biochar.

**Table 2:** Chlorophyll content in the leaves of alfalfa seedlings under different treatments.

Treatment	Chlorophyll a (mg·g^−1^)	Chlorophyll b (mg·g^−1^)	Total Chlorophyll (mg·g^−1^)
CK	1.46 ± 0.10 c	0.57 ± 0.02 b	2.03 ± 0.08 c
B1	1.82 ± 0.10 b	0.82 ± 0.04 a	2.64 ± 0.08 b
B2	2.02 ± 0.16 a	0.87 ± 0.06 a	2.89 ± 0.19 a
B3	1.94 ± 0.09 ab	0.83 ± 0.03 a	2.77 ± 0.10 ab

Note: Values represent mean ± SD. Different lowercase letters within the same column indicate statistically significant differences among treatments (*p* < 0.05). CK: Control treatment, B1: Treatment with 2.5% biochar, B2: Treatment with 5% biochar, B3: Treatment with 7.5% biochar.

**Table 3:** Nutritional composition of EMR under different treatments.

Treatment	pH	OM (g·kg^−1^)	AN (mg·kg^−1^)	AP (mg·kg^−1^)	AK (mg·kg^−1^)
CK	7.32 ± 0.03 d	9.12 ± 0.51 d	19.18 ± 0.89 d	6.19 ± 0.28 b	357.18 ± 22.07 a
B1	7.58 ± 0.07 c	10.51 ± 0.40 c	21.71 ± 1.79 c	6.99 ± 0.31 a	349.33 ± 11.28 a
B2	7.80 ± 0.03 b	12.14 ± 0.86 b	26.27 ± 1.06 a	6.94 ± 0.19 a	364.79 ± 12.25 a
B3	8.02 ± 0.12 a	13.34 ± 0.57 a	23.79 ± 0.99 b	6.38 ± 0.23 b	339.88 ± 23.98 a

Note: Values represent mean ± SD. Different lowercase letters within the same column indicate statistically significant differences among treatments (*p* < 0.05).EMR: Electrolytic manganese residue, OM: Organic matter, AN: Alkali-hydrolyzable nitrogen, AP: Available phosphorus, AK: Available potassium. CK: Control treatment, B1: Treatment with 2.5% biochar, B2: Treatment with 5% biochar, B3: Treatment with 7.5% biochar.

**Table 4:** The physicochemical properties of chili straw biochar and EMR.

Sample	pH	Organic Matter (g·kg^−1^)	Total Nitrogen (g·kg^−1^)	Alkaline Nitrogen (mg·kg^−1^)	Available Phosphorus (mg·kg^−1^)	Available Potassium (mg·kg^−1^)	Total Mn (mg·kg^−1^)	Available Mn (mg·kg^−1^)
EMR	7.46	5.17	0.45	18.96	6.83	385.49	41,052.67	644.84
Chili Straw Biochar	9.45	267.30	7.32	-	-	-	113.29	24.08

Note: Mn: Manganese. “-” indicates “not detected.”.

## Data Availability

The data presented in this study are available on request from the corresponding author. The data generated in this study are primarily comprised of routine soil chemical properties and plant growth and physiological parameters. These data have been presented in the manuscript as means ± standard deviations in the figures and tables. As the study did not involve raw sequencing data, genomic information, or species identification data that require public archiving, the original datasets were not deposited in a public repository. However, the corresponding author will gladly provide the relevant data upon reasonable request for academic purposes.

## Abbreviations

The following abbreviations are used in this manuscript:

Mn	Manganese
EMR	Electrolytic manganese residue
MDA	Malondialdehyde
AMn	Available Mn
GR	Germination rate
GE	Germination energy
GI	Germination index
OM	Organic matter
AN	Alkali-hydrolyzable nitrogen
AP	Available phosphorus
AK	Available potassium
